# Immunotherapy in Penile Squamous Cell Carcinoma: Present or Future? Multi-Target Analysis of Programmed Cell Death Ligand 1 Expression and Microsatellite Instability

**DOI:** 10.3389/fmed.2022.874213

**Published:** 2022-05-03

**Authors:** Marco Montella, Rosalaura Sabetta, Andrea Ronchi, Marco De Sio, Davide Arcaniolo, Ferdinando De Vita, Giuseppe Tirino, Alessandro Caputo, Antonio D’Antonio, Francesco Fiorentino, Gaetano Facchini, Giovanni Di Lauro, Sisto Perdonà, Jole Ventriglia, Gabriella Aquino, Florinda Feroce, Rodolfo Borges Dos Reis, Luciano Neder, Matteo Brunelli, Renato Franco, Federica Zito Marino

**Affiliations:** ^1^Pathology Unit, Department of Mental Health, Physic and Preventive Medicine University of Campania “Luigi Vanvitelli”, Naples, Italy; ^2^Urology Unit, Department of Woman Child and of General and Specialist Surgery, University of Campania “Luigi Vanvitelli”, Naples, Italy; ^3^Oncology Unit, Department of Precision Medicine, University of Campania “Luigi Vanvitelli”, Naples, Italy; ^4^Department of Medicine and Surgery, University Hospital “San Giovanni di Dio e Ruggi D’Aragona”, University of Salerno, Salerno, Italy; ^5^Department of Pathology, University Hospital “San Giovanni di Dio e Ruggi D’Aragona”, Salerno, Italy; ^6^Pathology Unit, S.M. delle Grazie Hospital, Pozzuoli, Italy; ^7^Medical Oncology Unit, S.M. delle Grazie Hospital, Pozzuoli, Italy; ^8^Urology Unit, S.M. delle Grazie Hospital, Pozzuoli, Italy; ^9^Department of Urogynecology, National Cancer Institute, Pascale Foundation (Scientific Institute for Research and Healthcare), Naples, Italy; ^10^Pathology Unit, Istituto Nazionale Tumori Fondazione G. Pascale IRCCS, Naples, Italy; ^11^Urology Division, Department of Surgery and Anatomy, Ribeirão Preto School Medicine, University of São Paulo, Ribeirão Preto, Brazil; ^12^Department of Pathology and Forensic Medicine, Ribeirão Preto Medical School, University of São Paulo, Ribeirão Preto, Brazil; ^13^Department of Pathology, University of Verona, Verona, Italy

**Keywords:** penile cancer, penile SCC, PD-L1, MSI, HPV, immunotherapy, squamous cell carcinoma

## Abstract

**Background:**

Penile cancer (PC) is an extremely rare malignancy, and the patients at advanced stages have currently limited treatment options with disappointing results. Immune checkpoint inhibitors anti-programmed cell death 1 (PD-1)/programmed cell death ligand 1 (PD-L1) are currently changing the treatment of several tumors. Furthermore, the microsatellite instability (MSI) and the deficient mismatch repair system (dMMR) proteins represent predictive biomarkers for response to immune checkpoint therapy. Until present, few data have been reported related to PD-L1 expression and MSI in PC. The main aim of our study was the evaluation of PD-L1 expression in tumor cells (TCs) and tumor-infiltrating lymphocytes (TILs) in immune cells and the analysis of dMMR/MSI status in a large series of PCs.

**Methods:**

A series of 72 PC, including 65 usual squamous cell carcinoma (USCC), 1 verrucous, 4 basaloid, 1 warty, and 1 mixed (warty-basaloid), was collected. Immunohistochemistry (IHC) was performed to assess PD-L1 expression using two different anti-PD-L1 antibodies (clone SP263 and SP142 Ventana) and MMR proteins expression using anti-MLH1, anti-PMS2, anti-MSH2, and anti-MSH6 antibodies. PCR analysis was performed for the detection of MSI status.

**Results:**

Of the 72 PC cases analyzed by IHC, 45 (62.5%) cases were TC positive and 57 (79%) cases were combined positive score (CPS) using PDL1 SP263. In our cohort, TILs were present in 62 out of 72 cases (86.1%), 47 (75.8%) out of 62 cases showed positivity to PDL1 clone SP142. In our series, 59 cases (82%) had pMMR, 12 cases (16.7%) had lo-paMMR, and only 1 case (1.3%) had MMR. PCR results showed that only one case lo-paMMR was MSI-H, and the case dMMR by IHC not confirmed MSI status.

**Conclusion:**

Our findings showed that PD-L1 expression and MSI status represent frequent biological events in this tumor suggesting a rationale for a new frontier in the treatment of patients with PC based on the immune checkpoint inhibitors.

## Introduction

Penile cancer (PC) is a disease with high morbidity and mortality. Its prevalence is relatively rare, occurring predominantly in elderly men; specifically, the mean age at diagnosis is 60 years with an age-related incidence rising constantly and reaching its highest level at 70 years. The worldwide variation of PC incidence is related to differences in socioeconomic and religious conditions: it constitutes up to 10% of malignant disease in men in some African, Asian, and South American countries, while in Western Europe and the United States, it represents about 0.6% of all malignancies. Poor penile hygiene, smegma retention, phimosis, and infection with human papillomavirus (HPV), mainly type 16, represent the major risk factors involved in PC pathogenesis (1).

The vast majority (almost 95%) of PCs include squamous cell carcinoma (SCC), being the usual keratinizing type the most common histotype. Other rare subtypes of SCC are basaloid (4%), warty (6%), mixed warty-basaloid (17%), verrucous (8%), papillary (7%), other SCC mixed (7%), and sarcomatoid carcinomas (1%) (2). Organ-preserving surgery with safety margins of not more than a few millimeters plus local radiotherapy is the current therapeutic standard for the early stages of the disease. Lymphogenic metastasis must be treated with radical lymphadenectomy and adjuvant chemotherapy, but, nevertheless, patients at advanced stages have currently limited treatment options with disappointing results (3).

Immunotherapy is gaining renewed interest as a treatment for cancer due to the promising clinical results observed with immune checkpoint inhibitors in several cancer types, such as non-small cell lung carcinoma (NSCLC) (4), melanoma (5), renal cell carcinoma, urothelial bladder cancer (6), head and neck SCC (7), and Hodgkin’s disease (8).

Programmed cell death 1 and ligand (PD-1/PD-L1) pathway represents one of the major immune checkpoint targets clinically investigated over the past few years. Currently, several clinical studies evaluating PD-1/PD-L1 inhibitors have been conducted in several different tumor types including breast, colorectal, anal, gastric, renal cell carcinoma, head, and neck, pancreatic, and hepatocellular cancer. The employment of immune checkpoint inhibitors has reported promising results consisting of increased survival and delayed tumor growth. Furthermore, to rationally design an ideal combination of cancer therapies based on tumor immunology, PD-L1 expression in TILs must also be evaluated (9).

Until present, few data have been reported related to PD-L1 expression in PC. The first report in 2016 revealed frequent PD-L1 expression in primary penile SCC unrelated to HPV status but associated with lymph node metastasis and shorter cancer-specific survival (10). More recently, some studies have found conflicting results about the prognostic value of PD-L1 expression in tumor cells (TCs) and/or TILs (11).

Another predictive biomarker for response to immune checkpoint therapy is MSI. The DNA mismatch repair (MMR) complex is a prominent cellular mechanism that protects cells from the accumulation of mutations occurring during DNA replication. The MMR system includes mainly four proteins (MLH1, PMS2, MSH2, and MSH6) that cooperatively detect and cut base-pair mismatches, leading to the consequent synthesis of the correct DNA strand.

Microsatellites are DNA sequences consisting of 1–6 repeated base pairs; their repetitive nature makes them prone to replication errors that are generally corrected by the MMR system (12). A deficient MMR system (dMMR) leads to MSI, a well-known sporadic event in tumors (10–15% of colorectal, gastric, and endometrial carcinomas), and the major background of hereditary non-polyposis colorectal cancer (HNPCC) syndrome. Recently, it has been shown that a dMMR system predicts a clinical benefit in response to immune checkpoint blockade therapy. The food and drug administration (FDA) approved the immune checkpoint inhibitors for the treatment of any solid cancer with a dMMR system and/or an MSI-high (MSI-H) genotype.

Until present, few data on the frequency of MSI and altered MMR protein expression are available for PC (13). In this context, we investigated the PD-L1 expression and the dMMR/MSI-H status in a large cohort of PCs could provide a rationale for a new frontier in the treatment of PC patients based on the immune checkpoint inhibitors.

## Materials and Methods

### Specimen

A series of 72 PC were included in our study. All cases were collected in our records at the University of Campania “L. Vanvitelli” Hospital, Fondazione G. Pascale, the University Ribeirão Preto Hospital of São Paulo, the Istituto Nazionale Tumori, the “Azienda Ospedaliera Universitaria Integrata Verona,” Verona (Italy), the S.M. delle Grazie Hospital, and the “AOU San Giovanni di Dio e Ruggi d’Aragona,” Salerno (Italy). The series included surgical samples and wide biopsies, as well as formalin-fixed paraffin-embedded (FFPE) samples. Sections of 4-μm thickness from each block (with a mean of 3 blocks per tumor) were stained with hematoxylin-eosin. All cases were reviewed according to the WHO histopathological classification (14).

### Immunohistochemistry Analysis of Programmed Cell Death Ligand 1 Expression

Immunohistochemistry (IHC) for PD-L1 was performed on 4-μm thick whole sections for each case on an automated Benchmark ULTRA staining platform (Ventana Medical Systems, Tucson, AZ, United States). The antibody clones used were SP263 and SP142 (Spring Biosciences, Pleasanton, CA, United States). Two independent observers (M.M. and R.F.) carried out IHC analysis; both observers were blinded.

Tumor cells or TILs with specific membranous and cytoplasmic staining were considered positive. Positive TCs and positive TILs were scored as the percentage of viable TCs (tumor proportion score) and the percentage of available TILs (TILs score).

To compare the clinical diagnostic performance of the assays, scores were dichotomized into positive or negative according to the clinically relevant cut-off values defined for the corresponding assay by the package inserts, FDA safety and effectiveness of datasheets, and the associated clinical trials of lung cancers for SP263 and breast cancers for SP142:

• SP263: < 1% of TCs positivity, between 1 and 50% of TCs positivity and > 50% of TCs positivity (15).
• SP142: > 1% of the tumor area occupied by PD-L1 positive lymphocytes and/or > 1% of TCs (16).

Moreover, SP263 PD-L1 expression in the tumor-associated mononuclear lymphocytes was scored using a combined positive score (CPS). The CPS was defined as the total number of TCs and TILs stained with PD-L1 divided by the number of all viable TCs, then multiplied by 100 (17), as applied for oral SCC. Each core contained at least 100 viable TCs.

### Immunohistochemistry Analysis of Mismatch Repair Protein Expression

Immunohistochemistry for four MMR proteins (MLH1, PMS2, MSH2, and MSH6) were performed on 4-μm thick whole sections for each case on an automated Benchmark ULTRA staining platform (Ventana Medical Systems, Tucson, AZ, United States). The antibody clones used were MLH1 (clone ES05, Agilent Technologies Canada Inc.), MSH2 (clone G219-1129, Becton Dickinson Biosciences, Canada), MSH6 (clone EPR3945, Abcam, Canada), and PMS2 (clone A16-4, BD Biosciences).

Adjacent normal tissue from each sample served as positive controls. MMR protein loss was defined by the absence of IHC staining in the nucleus of TCs while normal cells remained stained, ensuring the technical validity of the experiment. Immunohistochemical staining results were evaluated according to the scoring system reported in the literature: (i) proficient MMR (pMMR): cases with all four MMR staining positive; (ii) dMMR: cases with a loss of one of two heterodimers, including MLH1/PMS2 or MSH2/MSH6; we further considered another subset: (iii) cases with one MMR loss and/or the patchy expression of one or more MMR (lo-paMMR).

This heterogeneous patchy staining was defined according to the criteria established by Joost et al. as tumors show intra-tumor heterogeneity (strongly immunoreactive cells admixed with negative cells) and/or zonal loss (confluent areas of staining loss) (18).

In all cases labeled as showing MMR heterogeneity according to the patterns described above, there was a distinct loss of nuclear staining in tumor cells, while normal stroma and lymphocytes showed strong nuclear staining in the same areas, thus excluding artifact and/or staining failure. An arbitrary cut-off value of approximately 10% of the tumor showing either retention or loss of MMR proteins was used. All cases that showed a patchy expression and/or loss of MMR, they further analyzed by both IHC MMR and PCR on one or more tumor inclusions, if available. Two independent observers carried out the IHC analysis, both observers were blinded.

### PCR Detection for Microsatellite Status

Serial sections of 6-μm thickness from formalin-fixed paraffin-embedded matched normal and tumor tissues were routinely stained, and representative normal and tumor regions were identified by microscopic examination. Genomic DNA was isolated from the paraffin-embedded tissues using the QIAamp DNA mini kit (Qiagen, Valencia, CA) following the separation of tumor and normal tissue by manual microdissection. MSI was determined on tumor DNA using the EasyPGX^®^ ready MSI including the following mononucleotide repeats: BAT25, BAT26, NR21, NR22, NR24, NR27, CAT25, and MONO27; all data are summarized in Supplementary Table 1.

The test was performed according to the manufacturer’s instructions. PCR results were evaluated as follows: (i) microsatellite stable (MSS): cases with none of the markers unstable; (ii) MSI-H: tumor with 2 or more unstable markers; and (iii) MSI-L: cases with only one marker unstable; in these cases, new testing was carried out on non-tumor tissue, if available, to define a germinal mutation.

### Immunohistochemistry Analysis of p16 Expression

p16 IHC was carried out with a proprietary kit (CINtec Histology; MTM laboratories AG) using the clone E6H4 on a Ventana BenchMark automatic stainer (Ventana Medical Systems, Tucson, AZ, United States) for the detection of p16INK4a antigen. A PC with a high p16 expression was used as a positive control.

The primary antibody was omitted from negative controls. In our analysis, we identified the subgroups with different p16 IHC staining as follows:

1. p16 high expression: tumors with staining ≥ 70% nuclear and cytoplasmic staining;
2. p16 moderate expression: tumors with staining 30–70% nuclear and cytoplasmic staining;
3. p16 low expression: tumors with staining 10–30% nuclear and cytoplasmic staining;
4. p16 negative: tumors with staining 1–10% nuclear and cytoplasmic staining.

In addition, the intensity was also evaluated. The slides were independently evaluated by two separate observers.

### Automated Human Papillomavirus RNA *in situ* Hybridization

A section of 4-μm thickness from each case was used to perform the HPV RNA ISH test. Detection of high-risk HPV E6/E7 mRNA was performed using ready-to-use reagents from RNA scope 2.5 LS Reagent Kit-BROWN and the HPV-HR18 probe cocktail (Advanced Cell Diagnostics) that were loaded onto the Leica Biosystems’ BOND RX Research Advanced Staining System according to the user manual (Doc. No. 322,100-USM). The slides were independently evaluated by two separate observers.

Ubiquitin C and dapB were used as positive and negative controls, respectively. A positive HPV ISH test result was defined as positive if any of the malignant cells showed brown punctate dot-like nuclear and/or cytoplasmic positivity (19).

### Statistical Analysis

The Pearson χ^2^-test was used to determine the association of clinical characteristics with the status of protein expression. The Spearman rank test was used to assess the correlation between protein phenotypes. Statistical significance was set at the value of *p* = 0.05. Data analysis and summarization were carried out using SPSS 20.0 for Mac (SPSS Inc., Chicago, IL).

## Results

### Clinical and Pathological Features

In our series, patients older than 60 years were 45; the tumor site was the glans in 68 cases and the foreskin in 4. Usual squamous cell carcinoma (USCC) was observed in 65; a special histotype was diagnosed in 7 cases, particularly verrucous in 1 case, basaloid in 4 cases, warty in 1 case, and mixed warty-basaloid in 1 case. In the USCC histotype, the low grade (G1) was recorded in 18 cases, the moderate grade (G2) in 39 cases, and the high grade (G3) in 15 cases. Finally, the tumors were staged T1 in 24 cases, T2 in 25, T3 in 17, and T4 in 5, 1 case was *in situ* carcinoma.

p16 overexpression was recorded in 25 (34.7%) out of 72 cases, particularly 19 in USCC and 6 in special histotypes. p16 low-intensity expression was observed in 5 cases (7%), moderate-intensity expression in 6 cases (8%), and high-intensity expression in 13 cases (18%). HPV positive by ISH was observed in 13 (18%) out of 72 cases, particularly 9 in USCC and 4 in special histotypes. All data obtained are summarized in Table 1.

**TABLE 1:** Clinical and pathological features.

Features	TOTAL *n*. (%)	PD-L1 SP263 TPS		PD-L1 SP263 CPS		PD-L1 SP142			IHC MMRPs		
		Positive	Negative	Positive	Negative	TILs +	TILs -	NA	pMMR	lo-pa MMR	dMMR
**Mean age (years)**											
< 60	27 (37.5%)	20 (27.8%)	7 (9.7%)	22 (30.6%)	5 (7%)	19 (26.4%)	8 (11.1%)	/	19 (26.4%)	7 (9.7%)	1 (1.4%)
≥ 60	45 (62.5%)	25 (34.7%)	20 (27.8%)	35 (48.6%)	10 (13.9%)	28 (38.9%)	7 (9.7%)	10 (13.9%)	40 (55.6%)	5 (7%)	/
**Anatomical location**											
Glans	68 (94.4%)	41 (56.9%)	27 (37.5%)	53 (73.6%)	15 (20.8%)	43 (59.7%)	15 (20.8%)	10 (13.9%)	55 (76.4%)	12 (16.7%)	1 (1.4%)
Foreskin	4 (5.6%)	4 (5.6%)	/	4 (5.6%)	/	4 (5.6%)	/	/	4 (5.6%)	/	/
**T-STAGE**											
T1	24 (33.3%)	17 (23.6%)	7 (9.7%)	20 (27.8%)	4 (5.6%)	17 (23.6%)	5 (7%)	2 (2.8%)	23 (31.9%)	1 (1.4%)	/
T2	25 (34.7%)	14 (19.4%)	11 (15.3%)	19 (26.4%)	6 (8.3%)	14 (19.4%)	7 (9.7%)	4 (5.6%)	17 (23.6%)	7 (9.7%)	1 (1.4%)
T3	17 (23.6%)	10 (13.9%)	7 (9.7%)	13 (18%)	4 (5.6%)	12 (16.7%)	2 (2.8%)	3 (4.2%)	14 (19.4%)	3 (4.2%)	/
T4	5 (7%)	3 (4.2%)	2 (2.8%)	4 (5.6%)	1 (1.4%)	4 (5.6%)	/	1 (1.4%)	4 (5.6%)	1 (1.4%)	/
*In situ*	1 (1.4%)	1 (1.4%)	/	1 (1.4%)	/	/	1 (1.4%)	/	1 (1.4%)	/	/
**Hystological classification**											
USCC	65 (90.3%)	40 (55.6%)	25 (34.7%)	53 (73.6%)	12 (16.7%)	44 (61.1%)	12 (16.7%)	9 (12.5%)	52 (72.2%)	12 (16.7%)	1 (1.4%)
Verrucous	1 (1.4%)	1 (1.4%)	/	1 (1.4%)	/	1 (1.4%)	/	/	1 (1.4%)	/	/
Basaloid	4 (5.6%)	4 (5.6%)	/	3 (4.2%)	1 (1.4%)	2 (2.8%)	2 (2.8%)	/	4 (5.6%)	/	/
Warty	1 (1.4%)	/	1 (1.4%)	/	1 (1.4%)	/	/	1 (1.4%)	1 (1.4%)	/	/
Mixed (Warty-Basaloid)	1 (1.4%)	/	1 (1.4%)	/	1 (1.4%)	/	1 (1.4%)	/	1 (1.4%)	/	/
**Hystological grade**											
G1	18 (25%)	9 (12.5%)	9 (12.5%)	14 (19.4%)	4 (5.6%)	10 (13.9%)	5 (7%)	3 (4.2%)	14 (19.4%)	4 (5.6%)	/
G2	39 (54.2%)	26 (36.1%)	13 (18%)	33 (45.8%)	6 (8.3%)	28 (38.9%)	6 (8.3%)	5 (7%)	34 (47.2%)	4 (5.6%)	1 (1.4%)
G3	15 (20.8%)	10 (13.9%)	5 (7%)	10 (13.9%)	5 (7%)	9 (12.5%)	4 (5.6%)	2 (2.8%)	11 (15.3%)	4 (5.6%)	/
**P16 IHC**											
Positive	25 (34.7%)	17 (23.6%)	8 (11.1%)	19 (26.4%)	6 (8.3%)	14 (19.4%)	7 (9.7%)	4 (5.6%)	21 (29.2%)	4 (5.6%)	/
Negative	47 (65.3%)	28 (38.9%)	19 (26.4%)	38 (52.8%)	9 (12.5%)	33 (45.8%)	8 (11.1%)	6 (8.3%)	38 (52.8%)	8 (11.1%)	1 (1.4%)
**HPV ISH**											
Positive	13 (18%)	7 (9.7%)	6 (8.3%)	8 (11.1%)	5 (7%)	9 (12.5%)	1 (1.4%)	3 (4.2%)	10 (13.9%)	3 (4.2%)	/
Negative	59 (82%)	38 (52.8%)	21 (29.2%)	49 (68%)	10 (13.9%)	38 (52.8%)	14 (19.4%)	7 (9.7%)	49 (68%)	9 (12.5%)	1 (1.4%)
**TOTAL**	**72 (100%)**	45 (62.5%)	27 (37.5%)	57 (79.2%)	15 (20.8%)	47 (65.3%)	15 (20.8%)	10 (13.9%)	59 (82%)	12 (16.7%)	1 (1.4%)

*TPS, tumor proportion score; CPS, combined proportion score; IHC, immunohistochemistry; MMRPs, mismatch-repair proteins; pMMRPs, proficient mismatch-repair proteins; dMMRPs, deficient mismatch-repair proteins; lo-pa MMRPs, loss/patchy mismatch-repair proteins; TILs+, tumor-infiltrating lymphocytes present; TILs-, tumor-infiltrating lymphocytes absent; NA, Not Available; USCC, usual squamous cell carcinoma.*

### Immunohistochemistry Analysis of Programmed Cell Death Ligand 1 Expression

**FIGURE 1:**
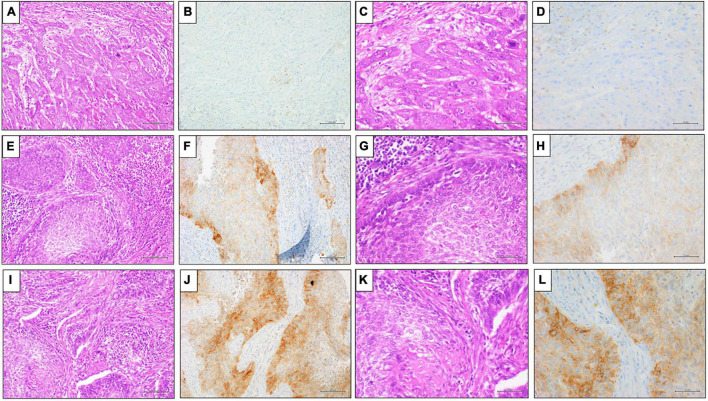
Representative results of PD-L1 (clone SP263) IHC expression by TPS in penile SCC. **(A,E,I)** Hematoxylin and Eosin (H&E) staining (20x magnification, scale bar 100 μm); **(C,G,M)** Hematoxylin and Eosin (H&E) staining (40x magnification, scale bar = 200 μm); **(B,D)** PD-L1 (clone SP263) IHC expression < 1%, DAB staining (40x magnification, scale bar = 200 μm); **(F,H)** PD-L1 (clone SP263) IHC expression 1% ≤ score < 50%, DAB staining (40x magnification, scale bar = 200μm); **(L,N)** PD-L1 (clone SP263) IHC expression ≥ 50%, DAB staining (40x magnification, scale bar = 200 μm).

**FIGURE 2:**
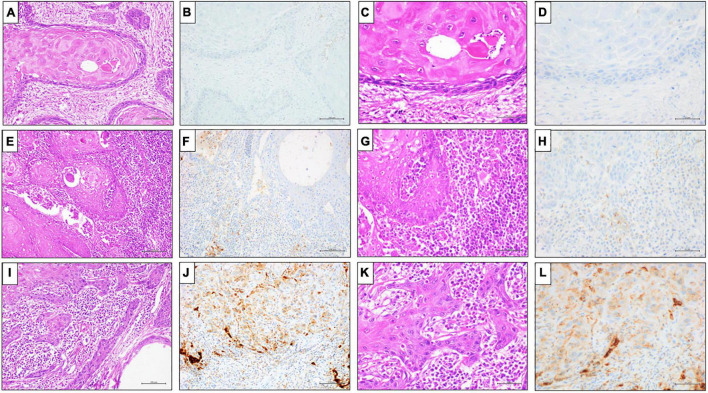
Representative results of PD-L1 (clone SP263) IHC expression by CPS in penile SCC. **(A,E,I)** Hematoxylin and Eosin (H&E) staining (20x magnification, scale bar = 100 μm); **(C,G,M)** Hematoxylin and Eosin (H&E) staining (40x magnification, scale bar = 200 μm); **(B,D)** PD-L1 (clone SP263) IHC expression score ≤ 1%, DAB staining (40x magnification, scale bar = 200 μm); **(F,H)** PD-L1 (clone SP263) IHC expression 1% < score < 20%, DAB staining (40x magnification, scale bar = 200 μm); **(L,N)** PD-L1 (clone SP263) IHC expression ≥ 20%, DAB staining (40x magnification, scale bar = 200 μm).

The penile SCCs showed variable positivity rates of PDL1 expression based on different clones and score applied, these data are summarized in Table 2. Of the 72 PC analyzed by PDL1 SP263 IHC, 45 (62.5%) cases showed positivity in TCs with different scores, particularly 27 cases (37%) showed TCs positivity < 1%, 15 cases (21%) showed TCs positivity between 1 and 50%, and 30 cases (42%) showed TCs positivity > 50% (Figure 1). PDL1 SP263 CPS evaluation showed 15 (21%) negative and 57 (79%) positive cases. Among these positive cases, 18 cases (25%) had a CPS between 1 and < 20, and 39 cases (54%) had a CPS > 20 (Figure 2).

**TABLE 2:** Results of the PD-L1 IHC evaluation.

PD-L1 evaluation			
	NEGATIVE N.cases (%)	POSITIVE N.cases (%)	
CPS score	<1 15 (21%)	1–≤ 20 18 (25%)	> 20 39 (54%)
TPS percentage	<1% 27 (37%)	1–≤ 50% 15 (21%)	> 50% 30 (42%)
TILs positivity	15 (24%)	47 (76%)	

*CPS, combined proportion score, TPS, tumor proportion score, TILs, tumor infiltrating lymphocytes.*

**FIGURE 3:**
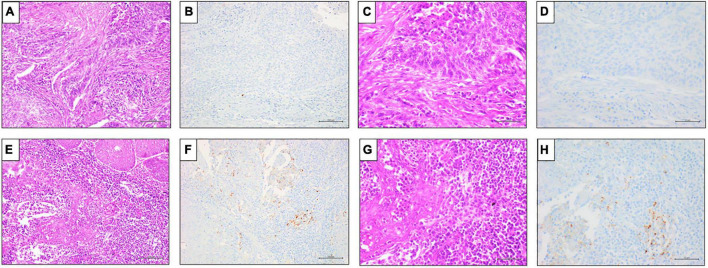
Representative results of PD-L1 SP142 IHC expression in TILs in penile SCC. **(A,E)** Hematoxylin and Eosin (H&E) staining (20x magnification, scale bar = 100 μm); **(C,G)** Hematoxylin and Eosin (H&E) staining (40x magnification, scale bar = 200 μm); **(B,F)** negative PD-L1 (clone SP142) IHC expression in TILs, DAB staining (40x magnification, scale bar = 200 μm); **(D,H)** positive PD-L1 (clone SP142) IHC expression in TILs, DAB staining (40x magnification, scale bar = 200 μm).

In our cohort, TILs were present in 62/72 (86.1%), 47 (75.8%) out of 62 cases showed positivity to PDL1 clone SP142 (Figure 3). Instead, as expected, only 7 (9.7%) out of 62 cases showed positivity to PDL1 clone SP142 also in TCs.

### Immunohistochemistry Analysis of Mismatch Repair Protein Expression

**FIGURE 4:**
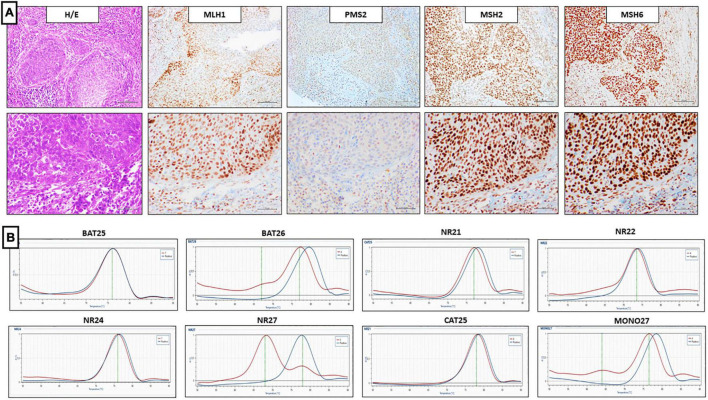
Representative results of penile SCC case showing lo-paMMR IHC and MSI-H by PCR. **(A)** Upper line (from left to right) MMR-IHC results: Hematoxilin and Eosin penile SCC, intact exspression of MLH1, MSH2 and MSH6, loss-patchy expression of PMS2 (20x magnification, scale bar = 100 μm); lower line (from left to right) MMR-IHC results: Hematoxilin and Eosin penile SCC, intact exspression of MLH1, MSH2 and MSH6, loss-patchy expression of PMS2 (40x magnification, scale bar = 200 μm). **(B)** MSI-PCR results: stability of BAT25, NR21, NR22, NR24 and CAT25, instability of BAT26, NR27, and MONO27 (red lines indicate samples while blue lines indicate MSS controls).

**FIGURE 5:**
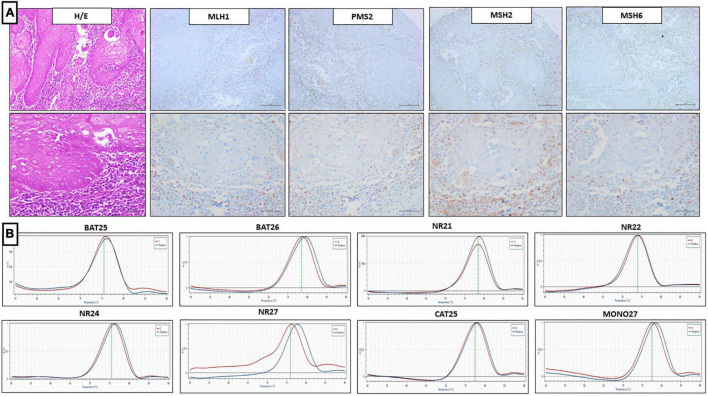
Representative results of penile SCC case showing dMMR IHC and MSI-L by PCR. **(A)** Upper line (from left to right) MMR-IHC results: Hematoxilin and Eosin penile SCC, loss expression of MLH1 and PMS2, while loss-patchy expression of MSH2 and MSH6 (20x magnification, scale bar = 100 μm); lower line (from left to right) MMR-IHC results: Hematoxilin and Eosin penile SCC, loss expression of MLH1 and PMS2, while loss-patchy expression of MSH2 and MSH6 (40x magnification, scale bar = 200 μm). **(B)** MSI-PCR results: stability of BAT25, BAT26, NR21, NR22, NR24, CAT25, and MONO27, instability of NR27 (red lines indicate samples while blue lines indicate MSS controls).

Of the 72 PC analyzed by IHC, there were 59 cases (82%) of pMMR, 12 cases (16.7%) of lo-paMMR (Figure 4), and only 1 case (1.3%) of dMMR (Figure 5). In detail, the dMMR case was negative for MLH1-PMS2. Among 12 cases of lo-paMMR, 7 cases showed the patchy expression of PMS2, 3 cases showed the patchy expression of two MMR, and 2 cases showed the patchy expression of MSH2 and loss of PMS2. These data are summarized in Table 3.

**TABLE 3:** Comparison of the results of MMR-IHC analysis and MSI-PCR analysis.

MMR-IHC		MSI-PCR		
		MSI-H	MSI-L	MSS
**n.1 dMMR**				
MLH1-PMS2	1		1	
**n.12 lo-paMMR**				
**n.3 patchy heterodimer**				
MLH1-PMS2	1			1
PMS2-MSH2	1			1
PMS2-MSH6	1			1
**n.7 patchy one MMR**				
PMS2	7	1		6
**n.2 loss one MMR-patchy**				
PMS2 lo and MSH2 P	2			2

### PCR Detection for Microsatellite Status

Of the 72 PC analyzed by IHC, all 13 cases harboring dMMR or lo-paMMR were analyzed by PCR. Among 13 cases, 11 cases were MSS, 1 case was MSI-H, and 1 case was MSI-L. In detail, MSI-H case showed the instability of BAT26, NR27, and MONO27 loci, while MSI-L showed the instability of NR27 only. Unfortunately, the testing on non-tumor tissue was not performed as normal tissue was not available. Furthermore, a series of 40 control cases that were determined as pMMR by IHC was analyzed using PCR, and all cases confirmed MSS status.

### Comparison of Mismatch Repair- Immunohistochemistry and Microsatellite Instability-PCR

Discordant results between IHC and PCR were obtained in penile SCC cases harboring dMMR or lo-paMMR. The case of dMMR by IHC resulted in MSI-L by PCR analysis. Among 12 cases of lo-paMMR, one case resulted in MSI-H and the remaining 11 cases resulted in MSS. These data are summarized in Table 3.

### Comparison of Programmed Cell Death Ligand 1 Immunohistochemistry and Microsatellite Instability Status

Comparing PD-L1 expression with the MMR IHC, different results were observed related to the different clones used in our analysis. In particular, using SP263 PD-L1 antibody, among 12 lo-paMMR cases, 7 cases were positive with both scores [tumor proportion score (TPS) and CPS] and 5 cases were negative. Using SP142 PD-L1 antibody, 6 cases were positive and 6 were negative. The case of dMMR was PD-L1 positive with both clones, particularly SP263 TPS was 40%, SP263 CPS was 60%, and SP142 was 20%.

The correlation between SP263 PD-L1 TCs expression > 1% and the dMMR/lo-paMMR by IHC was statistically significant (*p* < 0.005).

## Discussion

Squamous cell carcinoma of the penis is a rare and biologically aggressive malignancy, characterized by limited treatment options, not always curative, but just palliative in the advanced stage (20). Thus, new and efficient therapeutic strategies are needed to improve patient survival rates.

In recent years, immunotherapy has led to substantial changes in the cancer therapeutic paradigm. Nonetheless, the treatment with immune checkpoints showed relatively poor efficacy and low response rates in some tumors (21, 22). Therefore, adequate eligibility for immunotherapy requires the identification of biomarkers to distinguish the really sensitive patients and to predict response. To date, PD-L1 expression and dMMR/MSI-H represent the pivotal biomarkers to select the patients for immune checkpoint blockade therapies in the clinical practice of several tumors.

PD-L1 expression has been described in primary SCCs in different districts, including head and neck, lung and cervix (23, 24). Until present, PD1/PDL1 inhibitors were approved for the treatment of advanced SCCs, particularly two anti-PD-1 monoclonal antibodies, namely, Cemiplimab for advanced cutaneous SCC and nivolumab for metastatic or recurrent SCC of the head and neck (25, 26). Based on the histological and etiological similarities between SCCs regardless of the district, PDL1 could also play a role in penile SCC (10). Although several studies have investigated the PD-1/PD-L1 pathway in other urological malignancies, up to date, few data have been reported about PDL1 expression in penile SCC (10, 11, 27–29). In previous studies, several different clones, such as E1L3N, ZR3, 5H1, Dako 28.8, and SP142, were used to analyze PDL-1 positivity in penile SCC (10, 11, 20, 27–30). Davidsson et al. observed different percentages of PDL1-positive TCs according to the different clones used, particularly 32% of positive cases using clone 28.8 while 7.3% using clone SP142 (11). According to previous results, our data showed the PDL1 positivity in TCs in 44 cases (61%) using SP263 clone while in only 7 cases (9.7%) using SP142 clone.

The difference in PDL-1 expression in neoplastic cells when using different clones could be attributable to the specificity of the antibodies. Indeed, the SP263 clone is more specific for neoplastic cells, while the SP142 clone appears more specific for inflammatory cells (31). Particularly, SP263 was approved as the companion diagnostic for pembrolizumab in lung cancer patients, while SP142 was used for a clinical trial with atezolizumab in several cancer patients (32–34).

The FDA approved the treatment of triple-negative breast cancer (TNBC) with atezolizumab, basing the selection of patients on the SP142PDL1 expression in TILs of any intensity covering ≥ 1% of the tumor area (35).

In this context, the PDL1 expression in TILs must also be investigated in penile SCC to clarify its potential role as a predictive biomarker for the treatment with immune checkpoint inhibitors. Previous data reported the PDL1 immunohistochemical positivity in penile SCC in both TCs and TILs ranging between 32–62% and 64–80%, respectively (10, 11, 30).

In this study, the PD-L1 expression was evaluated on both TCs and TILs, using two different antibody clones, such as SP263 and SP142. In our series, approximately 75.8% of cases carrying TILs were PDL1 positive by IHC SP142. Our findings showed a higher rate of PDL1 positivity in TILs compared with previous reports, probably attributable to different methods for detection, including the choice of scoring method, the anti-PD-L1 antibody and/or cut-off value (11).

Different scoring systems have been currently proposed for PDL1 positivity in cancer cells based on the specific tumor (36). The scoring system applied in our study considers three tiers: positivity in cancer cells > 1%, positivity between 1 and 50%, and positivity > 50%. To date, this scoring system is used in clinical practice to select lung cancer patients for the treatment with pembrolizumab. Indeed, the patient is excluded from the treatment with pembrolizumab, when PDL-1 positivity is lower than 1%, the treatment in II line is considered if the PDL-1 positivity range is between 1 and 50% and finally the treatment in I line is successfully used when PDL-1 positivity is higher than 50% (37).

In penile SCC, there is no consensus about the PD-L1 IHC scoring system in previous reports. Thus, in some reports, only positive and negative are identified (27); in contrast, in some studies, 1 or 5% were considered the threshold values of positivity (10, 11, 20, 28, 30).

A CPS is an alternative method to TPS for scoring PD-L1 expression defined by the ratio of total positive tumor and immune to the total number of viable TCs. It is used in the characterization of several tumors, such as head and neck squamous cell carcinomas (HNSCC), to address patients to the immune checkpoint inhibitor pembrolizumab (38). In addition, the eligibility of the treatment with pembrolizumab is based on the usage of the CPS with different cut-off values according to the tumor type/site, such as gastric or gastroesophageal junction adenocarcinoma (CPS ≥ 1), locally advanced or metastatic urothelial carcinoma (CPS ≥ 10), recurrent or metastatic cervical cancer (CPS ≥ 1), recurrent locally advanced or metastatic SCC of the esophagus (CPS ≥ 10) (39), and recurrent or metastatic SCC of the head and neck (CPS ≥ 20) (17).

Muller et al. analyzed the expression of PDL1 (clone ZR3) in a series of 60 penile SCC HPV + (29). They found that 13% of cases showed TPS 0%, 47% TPS between 0 and 10%, and 40 TPS > 10%; about 3% had CPS score of 0, 33% had CPS score between 0 and 10, and 63% had CPS score of > 10.

In our study, we adopted the guidelines proposed in the HNSCC. Thus, CPS is > 1 in 79% (57/72) of cases, and a CPS is < 1 in 21% (15/72) of cases. Although CPS ≥ 50 seems to be an equivalent predictor to TPS ≥ 50% for selection of HNSCC patients potentially sensible to immune checkpoint inhibitor, based on our results, the CPS would allow to enrolling a greater number of patients than the TPS (21% CPS < 1 vs. 37% TPS < 1%). According to the previous data, CPS seems to be more reliable than TPS at lower cut-off values (CPS ≥ 1) in relation to the successful treatment, supporting the significance of PD-L1–positive immune cells as a sensitive biomarker (38).

To date, different cut-off values and scoring systems for PD-L1 evaluation have been validated in various tumor types. In this context, the definition of the PD-L1 IHC scoring system also in penile SCC represents an important turning point for an adequate selection of treatable patients. In particular, using CPS score could increase the percentage of PC patients potentially eligible for immunotherapy approximately by 16% compared with TPS score, since SCC is frequently characterized by PDL1 positivity of TILs.

Furthermore, no statistically significant data were reported in our series regarding the possible correlation of PD L1 expression with other clinical and pathological features. Previously, De Bacco et al. described, in a cohort of 40 penile SCC, a statistically significant association between p16 positivity and PD-L1 expression (clone ZR3) in tumors with worse clinical outcomes. On the contrary, in our PC series, no correlation between p16 expression and positive HPV ISH with PD-L1 expression was observed (28).

Besides PDL-1 expression, the defective DNA mismatch repair (MMR) system also predicted a clinical benefit in response to immunotherapy (28, 40). Usually, a proficient MMR system corrects the eventual presence of accumulated mutations, while a defective MMR system leads to global instability of repetitive sequences and coding regions. This phenomenon, called MSI, is already well known as a sporadic event in cancerogenesis, non-tumor specific (10–15% of colorectal, gastric, and endometrial carcinomas) (41). MSI can be molecularly categorized into two distinct phenotypes, namely, MSI-H and MSI-L (42).

Thus, the FDA approved the immunotherapy of any solid cancer with a defective MMR system and/or an MSI-H genotype (35). This approval led to a comprehensive investigation of MSI status across 39 cancer types, including bladder carcinoma, breast carcinoma, cervical SCC, diffuse large B-cell lymphoma, head and neck SCC, kidney renal clear cell carcinoma, lung adenocarcinoma, and more (43).

So far, in addition to this study, only a report of data of the MSI status in penile SCC has been published (13). Stoehr et al. analyzed the MSI status of 105 FFPE penile SCCs through PCR and the immunohistochemical expression of the MMR proteins. They found that 96 out of 105 cases provided interpretable results, but none of them showed MSI or loss of expression of the MMR proteins (13). Contrary to these results, in our series, we observed 1 case (1.3%) of dMMR and 12 cases (16.7%) of lo-paMMR. These discordant results could be due to technical limits, mainly linked to pre-analytical factors, especially tissue fixation. Moreover, Stoehr et al. have performed the study on TMA, unlike our study carried out on whole sections that have overcome the possible heterogeneity immunohistochemical expression of MMR proteins. Interestingly, the RT-PCR analysis showed MSI-H status of one case lo-paMMR suggesting that MSI-H could play a driver role in the development of penile SCC. Although MMR IHC represents a valid screening method, our data suggest that molecular tests could be performed in cases with doubtful IHC to avoid false-negative results. Moreover, both the PD-L1 and MMR expression could be affected by the intra-tumor heterogeneity that can lead to critical implications in the correct stratification of the patients to enroll for immunotherapy. In this view, technical and interpretative precautions must be used in the evaluation of these biomarkers.

Furthermore, our study showed that dMMR and lo-paMMR were statistically significantly associated with PD-L1 expression. Particularly, the case dMMR was PD-L1 positive with both clones regardless of the different scores used. Previous studies reported that PD-L1 + expression was closely related to dMMR/MSI-H status in other cancer types, particularly in colon-rectal cancers (4, 44, 45). Biologically, MSI-H tumors harbor a higher number of mutations in DNA coding sequences leading to increased production of the neoantigens and triggering immune activation (4, 46). For that reason, the association between the MSI-H status and PD-L1 expression could lead to increased sensitivity to immune checkpoint inhibitors due to an increased mutational burden of these tumors.

## Conclusion

Immunotherapies have led to a revolutionary change in the treatment of several cancer types; immune checkpoint blockade therapy would also be desirable in patients with penile SCC. However, the definitions of specific predictive biomarkers are needed to distinguish responders from non-responders patients. PDL1 expression and MSI status could represent the potential biomarkers in predicting immunotherapy efficacy in penile SCC. Further clinical trials using immune checkpoint blockade regimes in patients with PDL1 expression and MSI-H status may clarify the efficacy of immunotherapy and its possible clinical application in penile SCC.

## Data Availability Statement

The original contributions presented in the study are included in the article/Supplementary Material, further inquiries can be directed to the corresponding author/s.

## Ethics Statement

The studies involving human participants were reviewed and approved by the Università degli Studi della Campania L.Vanvitelli. The patients/participants provided their written informed consent to participate in this study.

## Author Contributions

All authors listed have made a substantial, direct, and intellectual contribution to the work, and approved it for publication.

## Conflict of Interest

The authors declare that the research was conducted in the absence of any commercial or financial relationships that could be construed as a potential conflict of interest. The handling editor AM declared a shared affiliation with the authors FFe, GA at the time of review.

## Publisher’s Note

All claims expressed in this article are solely those of the authors and do not necessarily represent those of their affiliated organizations, or those of the publisher, the editors and the reviewers. Any product that may be evaluated in this article, or claim that may be made by its manufacturer, is not guaranteed or endorsed by the publisher.
