# Supplementary material for: Immunotherapy in Penile Squamous Cell Carcinoma: Present or Future? Multi-Target Analysis of Programmed Cell Death Ligand 1 Expression and Microsatellite Instability
Source: Front Med (Lausanne). 2022 May 3;9:874213. doi: 10.3389/fmed.2022.874213 (PMC9113025; doi:10.3389/fmed.2022.874213)
Supplement: Supplementary file 1 [file Table_1.pdf]

Supplementary Table 1: Markers analyzed by EasyPGX© readyMSI kit.

marker	Gene	NCBI Ref. Seq.(GRCh 38.p12)	chromosome
BAT25	cKIT	NC_000004.12	4 (4q12)
BAT26	MSH2	NC_000002.12	2 (2p21-p16.3)
NR21	SLC7A8	NC_000014.9	14 (14q11.2)
NR22	STT3A	NC_000011.10	11 (11q24.2)
NR24	ZNF2	NC_000002.12	2 (2q11.1)
NR27	BIRC3	NC_000011.10	11 (11q22.2)
CAT25	CASP2	NC_000007.14	7 (7q34)
MONO27	MAP4K3	NC_000002.12	2 (2p22.1)
